# HOXA9 promotes homotypic and heterotypic cell interactions that facilitate ovarian cancer dissemination via its induction of P-cadherin

**DOI:** 10.1186/1476-4598-13-170

**Published:** 2014-07-14

**Authors:** Song Yi Ko, Honami Naora

**Affiliations:** 1 Department of Molecular & Cellular Oncology, University of Texas MD Anderson Cancer Center, Houston, TX 77030, USA

**Keywords:** Ovarian cancer, Homeobox gene, Metastasis, Cell adhesion, P-cadherin

## Abstract

**Background:**

Epithelial ovarian cancer (EOC) is a lethal disease that frequently involves the peritoneal cavity. Dissemination of EOC is a multi-step process in which exfoliated tumor cells survive in the peritoneal fluid as multi-cellular aggregates and then form invasive implants on peritoneal surfaces. The mechanisms that control this process are poorly understood. We previously identified that high expression of the developmental patterning gene *HOXA9* is associated with poor survival in EOC patients. In this study, we investigated the significance and mechanisms of *HOXA9* in controlling aggregation and implantation of floating EOC cells.

**Methods:**

HOXA9 was inhibited by shRNAs or expressed in EOC cells that were propagated in suspension cultures and in the peritoneal cavity of mice. Cell death was assayed by flow cytometry and ELISA. Cell aggregation, attachment and migration were evaluated by microscopy, transwell chamber assays and histopathologic analysis. DNA-binding of HOXA9 and its effect on expression of the cell adhesion molecule P-cadherin were assayed by chromatin immunoprecipitation, quantitative RT-PCR and Western blot. HOXA9 and P-cadherin expression was evaluated in publicly available datasets of EOC clinical specimens.

**Results:**

We identified that HOXA9 promotes aggregation and inhibits anoikis in floating EOC cells *in vitro* and in xenograft models. HOXA9 also stimulated the ability of EOC cells to attach to peritoneal cells and to migrate. HOXA9 bound the promoter of the *CDH3* gene that encodes P-cadherin, induced *CDH3* expression in EOC cells, and was associated with increased *CDH3* expression in clinical specimens of EOC. Inhibiting P-cadherin in EOC cells that expressed HOXA9 abrogated the stimulatory effects of HOXA9 on cell aggregation, implantation and migration. Conversely, these stimulatory effects of HOXA9 were restored when P-cadherin was reconstituted in EOC cells in which HOXA9 was inhibited.

**Conclusion:**

These findings indicate that HOXA9 contributes to poor outcomes in EOC in part by promoting intraperitoneal dissemination via its induction of P-cadherin.

## Background

More than 60% of women with epithelial ovarian cancer (EOC) are diagnosed with advanced-stage disease that has disseminated throughout the peritoneal cavity [1]. Despite advances in tumor debulking surgery and chemotherapy, patients with advanced-stage EOC have a 5-year survival rate of only 30% [1]. Whereas many other types of solid tumors metastasize via hematogenous or lymphatic routes, EOC cells typically spread by shedding into the peritoneal fluid which transports tumor cells, either as multi-cellular aggregates or as single cells, throughout the peritoneal cavity [2-4]. Subsequently, EOC cells attach to the mesothelium-lined peritoneal surfaces, such as the cavity wall, diaphragm and omentum, where they form invasive implants [2-4]. This ‘seeding’ of the peritoneal cavity with tumor cells is often associated with ascites formation. Although it is unclear whether EOC cells detach from the primary tumor as clusters or as single cells that then assemble into clusters, multi-cellular aggregates of floating EOC cells are increasingly regarded as ‘seeds of metastasis’ that are able to escape anoikis and implant on to peritoneal surfaces [3-5]. However, the tissue-specific mechanisms that facilitate the aggregation and implantation of floating EOC cells and drive the unique clinical behavior of this disease are poorly understood.

Homeobox genes encode transcription factors, commonly termed homeoproteins, that play essential roles in controlling developmental patterning and are expressed in a tightly regulated temporal and tissue-specific manner [6-8]. Many homeoproteins are aberrantly expressed in a variety of malignancies, but their functional significance in tumor progression is poorly understood as only few *bona fide* target genes have been identified [6,7]. The homeobox gene *HOXA9* is normally expressed during differentiation of the Müllerian ducts into the female reproductive tract [9]. We have identified that high *HOXA9* expression is strongly associated with poor overall survival of EOC patients [10]. Studies of mouse xenograft models revealed that expression of HOXA9 in EOC cells promotes growth of solid peritoneal implants by inducing normal peritoneal fibroblasts and mesenchymal stem cells to acquire features of cancer-associated fibroblasts that in turn supported tumor growth and angiogenesis [10]. This stimulatory effect of HOXA9 on solid tumor growth was attributed to its activation of the gene encoding transforming growth factor-β2 (TGF-β2) that acted in a paracrine manner on stromal cells [10]. Because EOC cells in solid tumors and in ascites have different biological behaviors and exist in different microenvironments, we investigated the possibility that HOXA9 mediates other types of effects in free-floating EOC cells. In this study, we identified that HOXA9 promotes the assembly of floating EOC cells into multi-cellular aggregates and inhibits anoikis, and also stimulates tumor-peritoneum interactions and tumor cell migration. These stimulatory effects of HOXA9 were found to be largely attributable to its induction of the cell adhesion molecule P-cadherin that is encoded by the *CDH3* gene, a transcriptional target of HOXA9.

## Results

### HOXA9 promotes aggregation and survival of floating EOC cells in i.p. xenograft models

**Figure 1:**
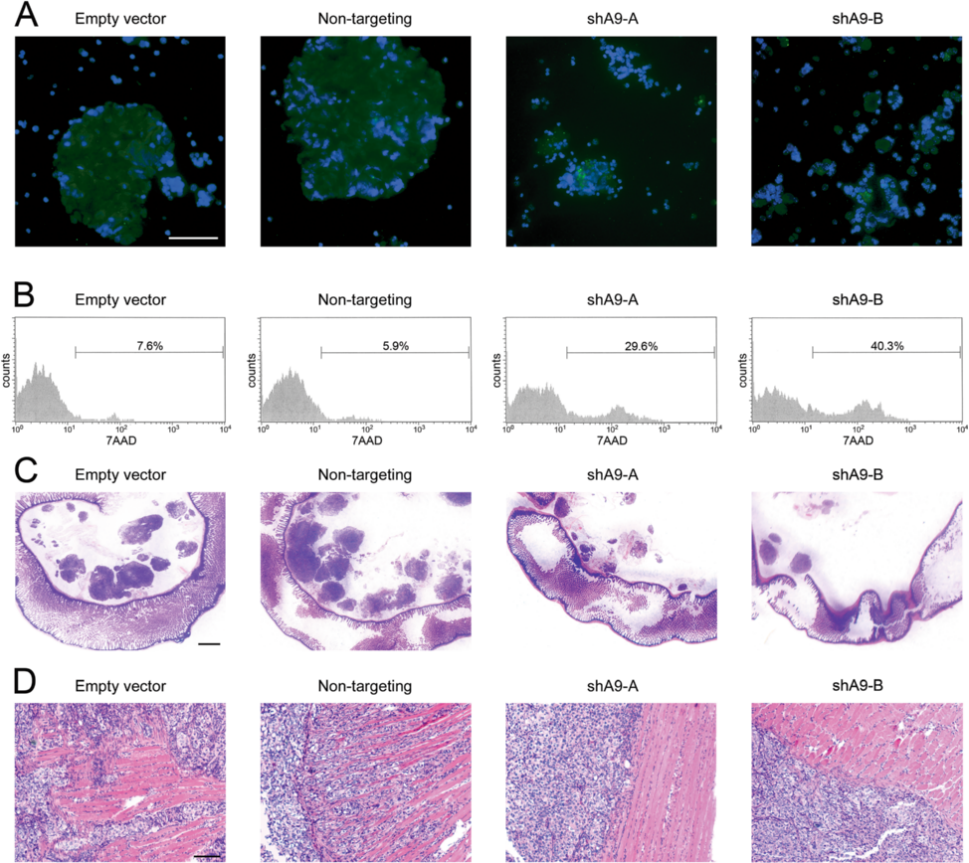
**Knockdown of HOXA9 inhibits aggregation and survival of ascitic EOC cells and decreases EOC cell implantation and invasiveness in i.p. xenograft models.** Female nude mice (n = 5 per group) were inoculated i.p. with equivalent numbers (2 × 10^6^) of GFP-expressing HOXA9+ control (empty vector, non-targeting) and HOXA9-knockdown (shA9-A, shA9-B) SKOV3ip cells. Mice were sacrificed at 3 weeks thereafter. **(A,B)** Ascitic cells were collected and stained with Hoechst dye to visualize nuclei and with 7AAD to assay cell death. **(A)** Representative examples of Hoechst staining (shown in blue) of ascitic cells where GFP-expressing tumor cells (shown in green) were visualized by fluorescence microscopy. Bar, 50 μm. **(B)** Flow cytometric analysis of 7AAD staining within the gated population of GFP + ascitic tumor cells. **(C,D)** Representative examples of HE-stained sections of **(C)** bowel tissues with mesenteric implants (bar, 1 mm) and **(D)** diaphragmatic implants (bar, 100 μm).

We previously identified that expression of HOXA9 in EOC cells promotes growth of solid tumor xenografts, but does not stimulate proliferation of EOC cells *in vitro*[10]. Because the biological behavior of ascitic tumor cells markedly differs from that of solid tumors, we investigated the effect of HOXA9 on floating EOC cells in ascites. I.p. xenograft models were generated from previously established HOXA9+ control and *HOXA9* shRNA-expressing SKOV3ip cell lines [10]. These EOC cell lines stably expressed GFP, enabling their detection among host cells in ascites. Floating EOC cells in ascites of control xenograft models (that expressed empty vector or non-targeting shRNA) were present as large compact aggregates [Figure 1A]. In contrast, ascites collected from HOXA9-knockdown models (shA9-A, shA9-B) contained smaller aggregates or single EOC cells [Figure 1A]. The aggregation of floating EOC cells is thought to enable these cells to escape anoikis [3,4]. Cell death was evaluated within the population of GFP-expressing ascitic EOC cells by flow cytometric analysis of 7-amino actinomycin D (7AAD) staining. As shown in Figure 1B, the proportion of ascitic EOC cells that exhibited cell death was substantially higher in the HOXA9-knockdown models than in control models. These observations raise the possibility that HOXA9 promotes aggregation of floating EOC cells and inhibits anoikis.

### HOXA9 also increases EOC cell implantation and invasiveness

Floating EOC cells that are transported by the peritoneal fluid frequently implant on the diaphragm, peritoneal cavity wall, omentum and mesentery [2-4]. We evaluated sections of tissues collected from mice that were inoculated with equivalent numbers of control and HOXA9-knockdown SKOV3ip cells. Whereas numerous mesenteric implants were detected in the control groups, fewer implants were detected in the HOXA9-knockdown groups [Figure 1C]. In EOC patients, implants tend to only invade the superficial bowel serosa and not the deeper layers [3]. Some superficial bowel serosa invasion was observed in control mice, but was markedly reduced in the HOXA9-knockdown groups (*P* < 0.05) [Additional file 1: Figure S1A]. Implants on the diaphragm extensively invaded adjacent muscle in the control groups, whereas invasive depth of diaphragmatic implants was significantly reduced in the HOXA9-knockdown groups (*P* < 0.01) [Figure 1D; Additional file 1: Figure S1B]. Similarly, implants on the peritoneal cavity wall were significantly less invasive in the HOXA9-knockdown groups than in the control groups (*P* < 0.01) [Additional file 1: Figure S1C]. These findings indicate that HOXA9 not only promotes aggregation and survival of floating EOC cells, but also increases the peritoneal implantation and invasiveness of these cells.

### HOXA9 promotes aggregation and inhibits anoikis of EOC cells in vitro

**Figure 2:**
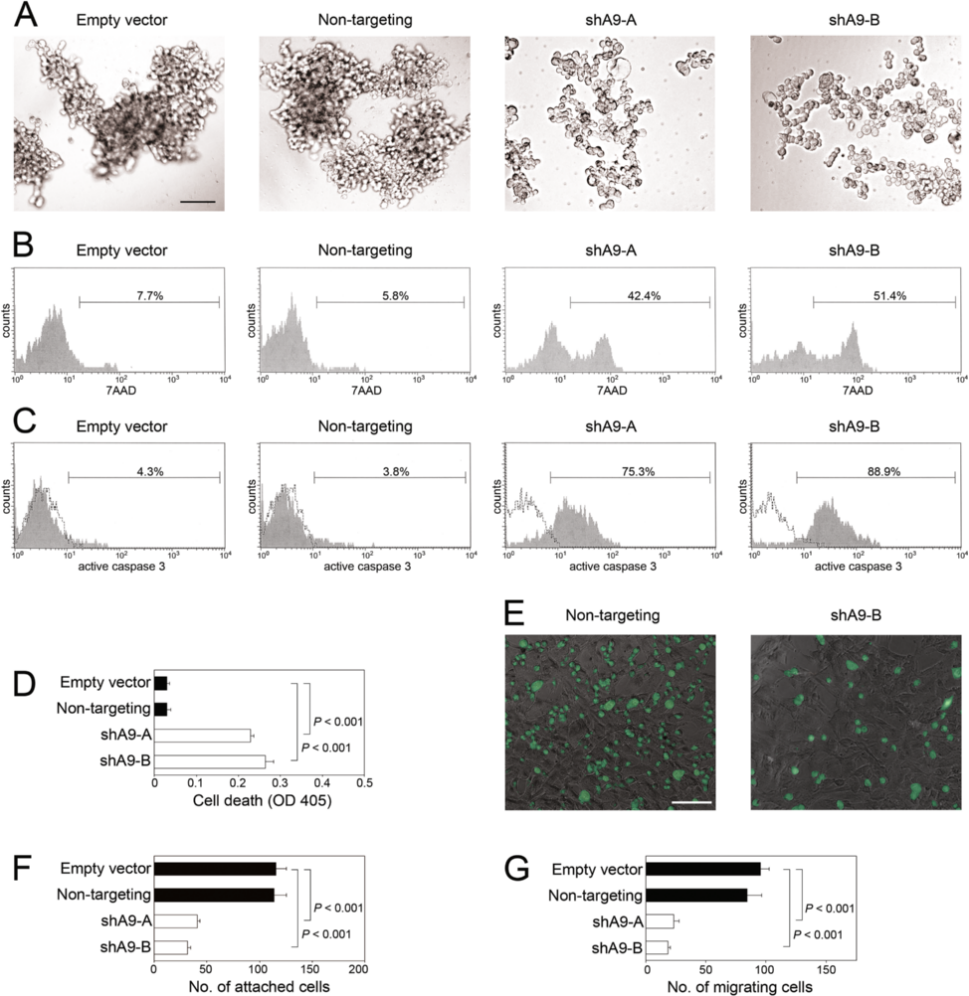
**Knockdown of HOXA9 inhibits aggregation of floating EOC cells, increases anoikis, decreases EOC-mesothelial cell interactions, and reduces EOC cell migration *in vitro. *(A-D)** Cells of control and HOXA9-knockdown SKOV3ip lines were incubated as suspension cultures in polyHEMA-coated plates for 3 days and evaluated for changes in cell morphology and cell death. **(A)** Cell morphology viewed by phase-contrast light microscopy. Bar, 100 μm. The extent of cell death was evaluated by flow cytometric analysis of **(B)** 7AAD staining and **(C)** staining of active caspase-3. In **(D)**, cell death was evaluated by assaying mono- and oligo- nucleosomes in cell lysates by ELISA. Shown are mean ± sd values of three independent experiments. Significance of differences was evaluated by Student *t-*test. **(E,F)** Equivalent numbers of control and HOXA9-knockdown SKOV3ip cells that stably expressed GFP were seeded onto confluent monolayers of normal human omental mesothelial cells. At 1 h thereafter, attached tumor cells were viewed by fluorescence microscopy and counted in three random 100× microscopic fields per assay. **(E)** Representative examples of SKOV3ip cells (shown in green) attached to mesothelial monolayers. Bar, 100 μm. **(F)** Mean ± sd values of three independent attachment assays. **(G)** Equivalent numbers of control and HOXA9-knockdown SKOV3ip cells were seeded in transwell chambers. At 6 h thereafter, migrating cells were counted in three random 100x microscopic fields per assay. Shown are mean ± sd values of three independent migration assays.

The development of tumor implants at distal sites depends on the survival of free-floating EOC cells that escape anoikis by forming multi-cellular aggregates [3,4]. We investigated the possibility that HOXA9 promotes aggregation and survival of floating EOC cells in *in vitro* assays, independently of implantation and of effects of host cells. Cells of control and HOXA9-knockdown SKOV3ip lines were incubated as suspension cultures in plates coated with poly(2-hydroxyethyl methacrylate) (polyHEMA), an inert polymer that prevent cells from adhering to substratum. Whereas control cells formed large aggregates, suspension cultures of HOXA9-knockdown cells comprised of single cells and small aggregates of loosely clustered cells [Figure 2A]. Cell death was observed in HOXA9-knockdown cells as detected by staining with 7AAD [Figure 2B]. In contrast, control cells were mostly viable [Figure 2B]. Evaluation of active caspase 3 levels indicated higher levels of apoptotic cell death in HOXA9-knockdown cells than in control cells [Figure 2C]. The increased level of cell death in HOXA9-knockdown cells was additionally confirmed by assaying mono- and oligo- nucleosomes in cell lysates [Figure 2D]. Together, these observations indicate that HOXA9 promotes the assembly of floating EOC cells into multi-cellular aggregates and inhibits anoikis.

### HOXA9 stimulates interactions between EOC cells and peritoneal mesothelial cells and promotes EOC cell migration in vitro

In subsequent experiments, we performed short-term *in vitro* attachment assays to evaluate the effect of HOXA9 on interactions between EOC cells and peritoneal mesothelial cells independently of its effects on aggregation and survival of EOC cells. Equivalent numbers of control and HOXA9-knockdown SKOV3ip cells were seeded as single cell suspensions onto confluent monolayers of normal human omental mesothelial cells. Attachment of SKOV3ip cells to mesothelial cells was assayed at 1 h after seeding during which time no significant change in cell aggregation or survival occurred. The numbers of HOXA9-knockdown SKOV3ip cells that bound to mesothelial cells were significantly lower than the numbers of bound control SKOV3ip cells (*P* < 0.001) [Figures 2E,F]. To evaluate the effect of HOXA9 on the migratory potential of EOC cells independently of its effects on cell aggregation and mesothelial attachment, we assayed migration of EOC cells that were seeded as single cell suspensions into transwell chambers. As compared to control SKOV3ip cells, HOXA9-knockdown SKOV3ip cells exhibited significantly reduced migration (*P* < 0.001) [Figure 2G]. These findings indicate that HOXA9 expression in EOC cells stimulates EOC-mesothelial cell interactions and EOC cell migration, and that these stimulatory effects are independent of its effect on EOC cell aggregation and survival.

### HOXA9 binds the CDH3 promoter and induces P-cadherin expression

**Figure 3:**
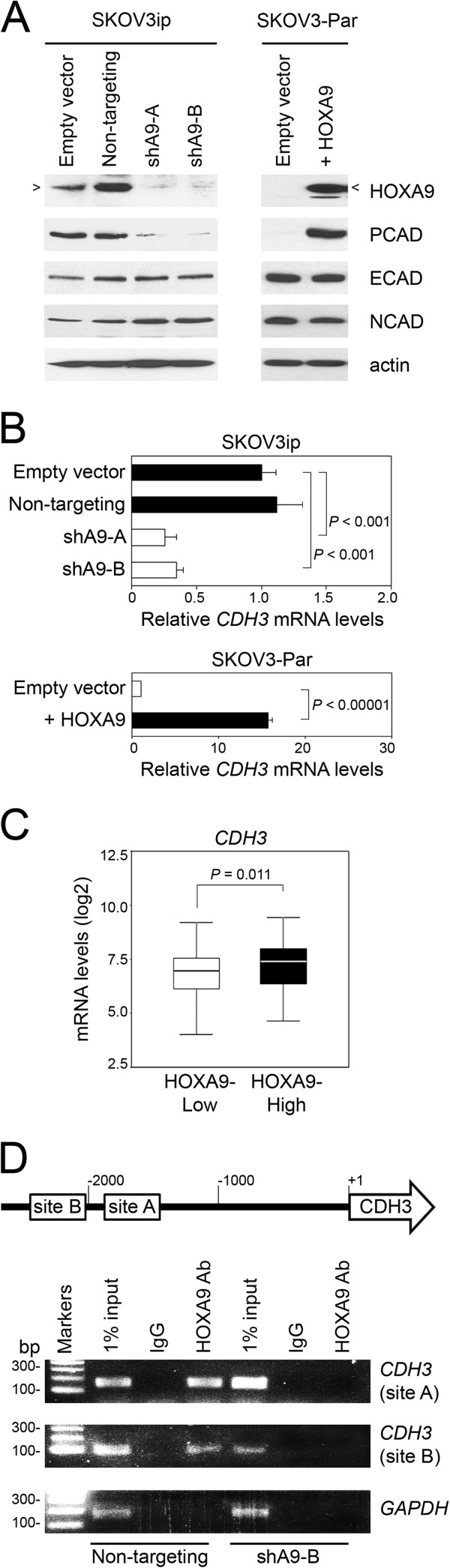
**HOXA9 induces P-cadherin expression in EOC cells. (A)** Western blot analysis of HOXA9 and cadherin levels in control and HOXA9-knockdown SKOV3ip lines and in vector-control and HOXA9-transfected SKOV3-Par lines. The 34 kD band corresponding to HOXA9 is indicated by an arrow. **(B)** qRT-PCR analysis of relative *CDH3* mRNA levels in SKOV3ip and SKOV3-Par lines. Significance of differences in *CDH3* mRNA levels was evaluated by Student *t*-test. **(C)** EOC cases from the TCGA Project (n = 567) were stratified according to *HOXA9* expression in tumors, where *HOXA9* mRNA levels were defined as High (≥ upper quartile) and Low (≤ lower quartile). Significance of differences in *CDH3* mRNA levels (log2 scale) between upper and lower quartile sub-groups was evaluated by Mann–Whitney *U*-test. **(D)** Schematic representation of the human *CDH3* promoter showing locations of the two HOXA9-binding sites (site A: 5′-TCATTTAAAAC-3′ and site B: 5′-TAATTTATTTAATAC-3′). Binding of endogenous HOXA9 in control SKOV3ip cells to these sites was detected by chromatin IP. Negative controls included IP using cells expressing *HOXA9* shRNA (shA9-B) and IP with IgG. *GAPDH* was amplified as an irrelevant gene control.

The classical cadherin P-cadherin has been reported to be induced during early stages of EOC progression and to promote EOC cell migration [11-13]. We recently identified that P-cadherin also facilitates aggregation of floating EOC cells, prevents anoikis and mediates the attachment of EOC cells to peritoneal mesothelial cells [14]. Because HOXA9 is a transcription factor, we investigated the possibility that HOXA9 induces P-cadherin expression. P-cadherin levels were markedly reduced when HOXA9 was knocked-down in SKOV3ip cells [Figure 3A]. Quantification of this protein down-regulation is shown in Additional file 2: Figure S2A. The down-regulation of P-cadherin was confirmed by quantitative reverse transcription PCR (qRT-PCR) analysis of transcript levels of *CDH3*, the gene encoding P-cadherin [Figure 3B]. Knockdown of HOXA9 in SKOV3ip cells did not down-regulate levels of other classical cadherins [Figure 3A]. SKOV3ip is an aggressive subclone that originally derived from the parental SKOV3 cell line (SKOV3-Par) [15]. In contrast to SKOV3ip cells, SKOV3-Par cells did not endogenously express HOXA9 or P-cadherin [Figure 3A]. Stable expression of HOXA9 in SKOV3-Par cells induced P-cadherin expression at the protein and mRNA levels [Figures 3A,B]. The Cancer Genome Atlas (TCGA) dataset is the largest compilation of gene expression data of clinical specimens of EOC. To determine whether P-cadherin expression is elevated in clinical specimens of EOC that highly express HOXA9, we stratified EOC cases in the TCGA dataset (n = 567 cases) into quartile sub-groups according to the levels of *HOXA9* transcripts of tumors. As shown in Figure 3C, levels of *CDH3* transcripts were found to be significantly higher in *HOXA9*-High tumors (upper quartile sub-group) than in *HOXA9*-Low tumors (lower quartile sub-group) (*P* = 0.011). Two putative consensus HOXA9-binding sites were identified in the *CDH3* promoter at 1.8 kb (site A) and at 2.1 kb (site B) upstream of the transcription start site [Figure 3D]. Binding of endogenous HOXA9 to both sites was detected by chromatin immunoprecipitation (IP) assays in control SKOV3ip cells, but no binding was detected in HOXA9-knockdown SKOV3ip cells [Figure 3D]. These findings indicate that HOXA9 induces P-cadherin expression and that the *CDH3* gene is a transcriptional target of HOXA9.

### P-cadherin inhibition abrogates the stimulatory effects of HOXA9 on EOC cell aggregation, implantation and migration

**Figure 4:**
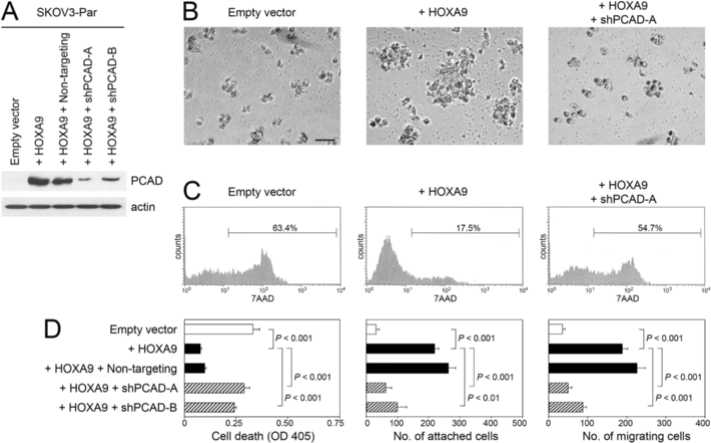
**Inhibition of P-cadherin in HOXA9-overexpressing EOC cells abrogates the stimulatory effects of HOXA9 on cell aggregation, survival, implantation and migration. (A)** Western blot analysis of P-cadherin levels in vector-control and HOXA9-transfected SKOV3-Par lines, and in HOXA9-transfected SKOV3-Par lines that stably expressed non-targeting shRNA and shRNAs targeting two different sites within the *CDH3* gene (shPCAD-A, shPCAD-B). **(B,C)** Cells of SKOV3-Par lines were incubated as suspension cultures in polyHEMA-coated plates for 3 days. **(B)** Cell morphology viewed by phase-contrast light microscopy. Bar, 50 μm. **(C)** Evaluation of cell death by flow cytometric analysis of 7AAD staining. **(D)** Evaluation of cell death by ELISA in suspension cultures of SKOV3-Par cells, attachment of SKOV3-Par cells to confluent mesothelial cell monolayers, and migration of SKOV3-Par cells were assayed as described in Figures 2D, E and G, respectively. Shown in D are mean + sd values of three independent assays.

To determine whether HOXA9 promotes EOC cell aggregation, implantation and migration by inducing P-cadherin expression, we evaluated the effects of inhibiting P-cadherin in EOC cells that expressed HOXA9. P-cadherin levels that were induced by enforced HOXA9 expression in SKOV3-Par cells were knocked-down by shRNAs targeting two different sites within the *CDH3* gene (shPCAD-A, shPCAD-B) [Figure 4A]. Quantification of this knockdown is shown in Additional file 2: Figure S2B. Knockdown of P-cadherin in HOXA9+ SKOV3-Par cells reduced cell aggregation [Figure 4B]. Cell death in HOXA9+ SKOV3-Par cells was significantly increased when P-cadherin was knocked-down (*P* < 0.001) [Figures 4C,D]. To confirm our findings, we evaluated the effects of inhibiting P-cadherin by a neutralizing antibody (Ab). Treatment of suspension cultures of HOXA9+ SKOV3-Par cells with P-cadherin Ab inhibited cell aggregation and increased cell death, as compared to cells that were treated with control IgG [Additional file 3: Figure S3A,B]. In addition, knockdown of P-cadherin in HOXA9+ SKOV3-Par cells significantly reduced the ability of these tumor cells to attach to mesothelial cells (*P* < 0.01) and to migrate (*P* < 0.001) [Figure 4D]. Together, these observations indicate that the stimulatory effects of HOXA9 on aggregation and survival of floating EOC cells, mesothelial attachment and EOC cell migration are largely mediated by its induction of P-cadherin expression.

### Stimulatory effects of HOXA9 on EOC cell aggregation, implantation and migration are recapitulated by P-cadherin in vitro and in vivo

**Figure 5:**
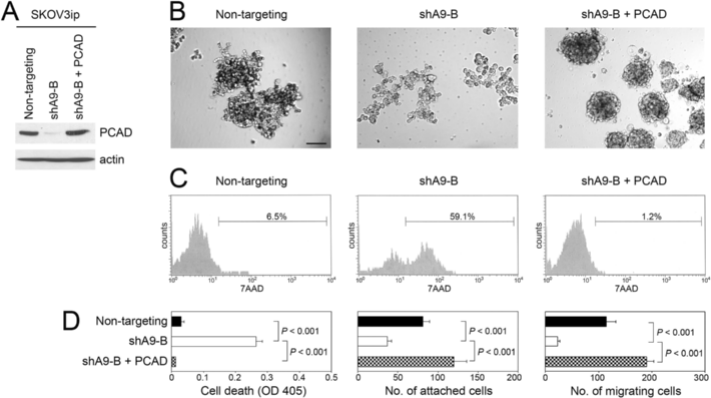
**Reconstitution of P-cadherin in HOXA9-knockdown EOC cells restores the stimulatory effects of HOXA9 on cell survival, implantation and migration *in vitro. *(A)** Western blot analysis of P-cadherin levels in HOXA9+ control SKOV3ip cells (non-targeting), HOXA9-knockdown SKOV3ip cells (shA9-B) and HOXA9-knockdown SKOV3ip cells that stably expressed P-cadherin (shA9-B + PCAD). **(B)** Morphology of SKOV3ip cells following incubation as suspension cultures in polyHEMA-coated plates for 3 days. Bar, 100 μm. **(C)** Evaluation of cell death in suspension cultures by flow cytometric analysis of 7AAD staining. **(D)** Evaluation of cell death by ELISA in suspension cultures of SKOV3ip cells, attachment of SKOV3ip cells to confluent mesothelial cell monolayers, and migration of SKOV3ip cells. Shown in D are mean + sd values of three independent assays.

In converse experiments, we evaluated whether the stimulatory effects of HOXA9 on EOC cell aggregation, implantation and migration can be restored when P-cadherin is reconstituted in EOC cells in which HOXA9 is inhibited. *CDH3* cDNA was stably expressed in HOXA9-knockdown SKOV3ip cells (shA9-B + PCAD) to restore the level of P-cadherin that was similar to the endogenous P-cadherin level in HOXA9+ control (non-targeting) SKOV3ip cells [Figure 5A, Additional file 2: Figure S2C]. Reconstitution of P-cadherin in HOXA9-knockdown SKOV3ip cells markedly increased cell aggregation and significantly decreased cell death in suspension cultures (*P* < 0.001) [Figures 5B-D]. Reconstitution of P-cadherin also increased the ability of HOXA9-knockdown SKOV3ip cells to attach to mesothelial monolayers and to migrate in *in vitro* assays [Figure 5D].

**Figure 6:**
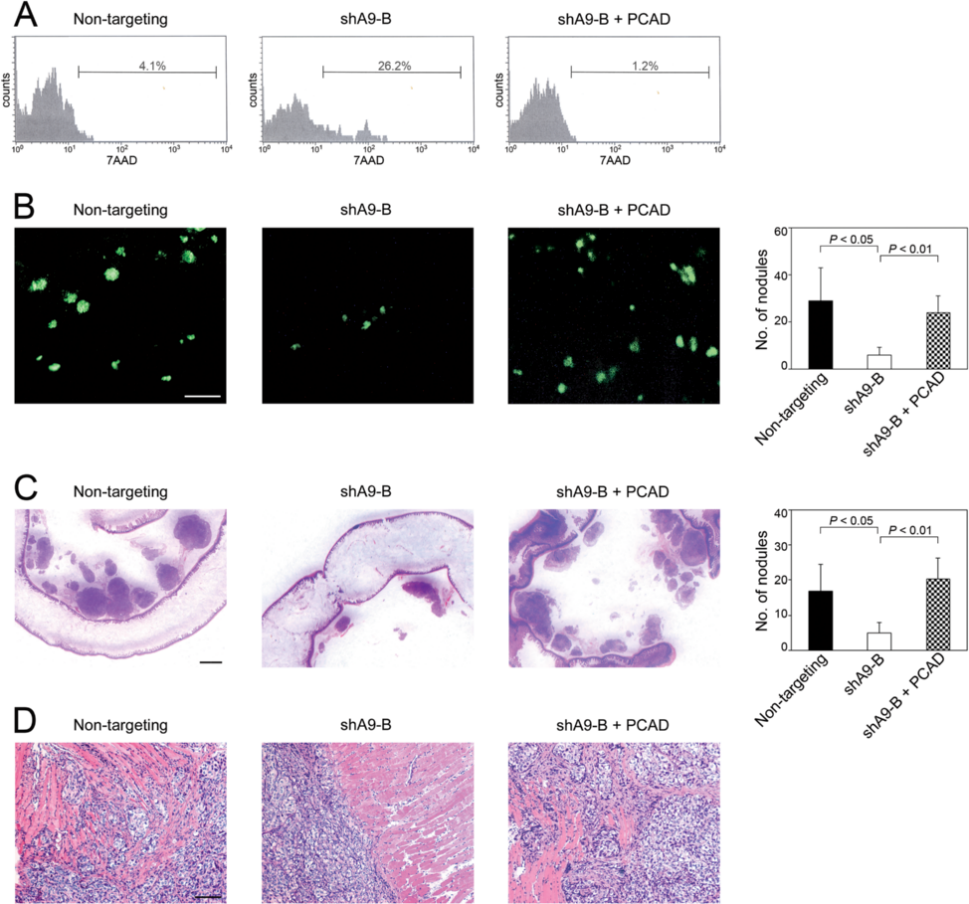
**Reconstitution of P-cadherin in HOXA9-knockdown EOC cells restores the stimulatory effects of HOXA9 on cell survival, implantation and migration *in vivo.*** Female nude mice were inoculated i.p. with equivalent numbers (2 × 10^6^) of cells of control (non-targeting) and HOXA9-knockdown (shA9-B) SKOV3ip lines and HOXA9-knockdown SKOV3ip cells that stably expressed P-cadherin (shA9-B + PCAD). In **(A)**, mice were sacrificed at 3 days following tumor cell inoculation. Floating cells in the peritoneal cavity were collected and stained with 7AAD. Cell death within the gated population of GFP+ tumor cells was evaluated by flow cytometric analysis of 7AAD staining. In **(B-D)**, mice were analyzed at 3 weeks following tumor cell inoculation. **(B)** Representative examples of GFP-expressing tumor implants (shown in green) attached to the peritoneal cavity wall as viewed by fluorescence microscopy. Bar, 1 mm. Numbers of implants on the peritoneal cavity wall were counted in five random 25 mm^2^ fields per mouse. **(C)** Representative examples of HE-stained sections of bowel tissues with mesenteric implants. Bar, 1 mm. Numbers of mesenteric implants were counted in three random 1 cm^2^ fields per mouse. **(D)** Representative examples of HE-stained sections of diaphragmatic implants. Bar 100 μm.

To confirm the findings of our *in vitro* studies, we firstly evaluated anoikis in floating EOC cells in short-term *in vivo* assays. Based on our prior experience using i.p. xenograft models derived from SKOV3ip cells [10,14], the earliest time-point at which solid peritoneal implants can be detected in mice is approximately 10 days following tumor cell inoculation. To assay anoikis in floating EOC cells prior to implantation, groups of mice were inoculated with equivalent numbers of non-targeting, shA9-B and shA9-B + PCAD SKOV3ip cells. At 3 days thereafter, GFP-expressing EOC cells in the peritoneal fluid were evaluated for cell death. The proportion of floating EOC cells that exhibited cell death in the shA9-B + PCAD model was markedly lower than that in the shA9-B model and similar to that in the control model [Figure 6A]. The numbers of implants that subsequently developed on the peritoneal cavity wall in the shA9-B + PCAD group were significantly higher than the numbers of implants in the shA9-B group (*P* < 0.01) and almost equivalent to the numbers of implants in the control group [Figure 6B]. Similar results were obtained when we evaluated the numbers of mesenteric implants in these groups of mice [Figure 6C]. Tumor implants that derived from shA9-B + PCAD cells and formed on the diaphragm, bowel serosa and cavity wall were found to be as invasive as control tumors and to exhibit greater invasive depth than implants that derived from shA9-B cells and formed at the same sites [Figure 6D; Additional file 1: Figures S1A-C]. Together, our findings indicate that HOXA9 promotes the aggregation and survival of floating EOC cells and also the peritoneal attachment and invasiveness of EOC cells, and that these stimulatory effects of HOXA9 are largely due to its induction of P-cadherin expression.

## Discussion

Substantial evidence indicates that aberrations in signaling pathways that control normal developmental patterning play pivotal roles in driving tumorigenesis [16-18]. Homeoproteins comprise another important class of patterning regulators and have been increasingly found to be aberrantly expressed in a variety of cancers [6,7]. Several homeoproteins promote tumor cell proliferation by deregulating transcription of genes that control cell cycle progression and autocrine growth-stimulatory pathways [19-21]. There is also evidence that some homeoproteins promote metastasis. For example, SIX1 induces epithelial-to-mesenchymal transition (EMT) in breast cancer cells by activating transcription of the gene encoding the TGF-β type I receptor [22] and promotes metastasis of rhabdomyosarcoma by inducing expression of the cytoskeletal protein ezrin [23]. Both normal patterning and tumor progression involve dynamic changes in homotypic and heterotypic cellular interactions, but surprisingly only few genes that control cell adhesion have been identified as homeoprotein targets [24,25]. We previously identified that high *HOXA9* expression is strongly associated with poor overall survival in EOC patients and promotes tumor progression in EOC xenograft models [10]. The findings of this present study indicate that the *CDH3* gene which encodes P-cadherin is a transcriptional target of HOXA9 and that HOXA9 promotes both homotypic and heterotypic cell interactions that facilitate intraperitoneal dissemination of EOC through its induction of P-cadherin.

The propensity for intraperitoneal ‘seeding’ is a hallmark of EOC and depends on adaptation by EOC cells to two dynamic changes in their microenvironment. The first change occurs when EOC cells are exfoliated and transported by the flow of the peritoneal fluid. It is widely thought that aggregation enables floating EOC cells to escape anoikis [3,4], but the mechanisms that facilitate this aggregation are poorly understood. The present study indicates that HOXA9 promotes aggregation of floating EOC cells by inducing expression of P-cadherin. Inhibiting P-cadherin in HOXA9+ EOC cells abolished the stimulatory effect of HOXA9 on aggregation, whereas aggregation was almost completely restored when P-cadherin was reconstituted in EOC cells in which HOXA9 was inhibited. HOXA9 had little effect on levels of other classical cadherins, but it is not possible to exclude the possibility that HOXA9 might also promote tumor cell aggregation by inducing expression of other types of adhesion molecules. The classical cadherins have well-characterized functions in facilitating homotypic cell adhesion [26,27]. The significance of P-cadherin in mediating aggregation of floating EOC cells is supported by our recent study in which we demonstrated that inhibiting P-cadherin reduces aggregation of ascitic EOC cells in xenograft models [14]. High expression of P-cadherin has been found to be associated with poor overall survival of EOC patients [12]. A study by Patel and colleagues identified that P-cadherin is the predominant type of cadherin expressed in tumor cells in the peritoneal fluid of EOC patients [11]. Sivertsen and colleagues reported that P-cadherin is frequently expressed in effusion specimens of EOC patients (51 of 53 cases), but also found that E-cadherin and N-cadherin are expressed at similar frequency [28]. There is discordance in the literature regarding the expression of E-cadherin and N-cadherin in effusion specimens of EOC patients. Two studies have reported that levels of E-cadherin and N-cadherin are decreased in effusions as compared to solid tumors [11,29], whereas a third study identified that E-cadherin levels were higher in effusions than in matching primary solid tumors [30].

Although it is widely thought that aggregation enables floating tumor cells to escape anoikis, the function of cadherins in anoikis appears to be cell-specific. Inhibition of E-cadherin increases anoikis sensitivity in Ewing sarcoma cells [31], but increases anoikis resistance in mammary epithelial cells [32]. Our recent studies support the notion that P-cadherin primarily inhibits anoikis in EOC cells by promoting cell aggregation. Inhibition of P-cadherin increased cell death in suspension cultures but not in adherent cultures of EOC cells, and increased cell death in ascitic EOC cells but not in solid tumor xenografts [14]. The possibility that P-cadherin might promote cell survival independently of cell aggregation cannot be excluded. Cell adhesion is facilitated by interactions between the extracellular domains of cadherin molecules on adjacent cells [26]. Intracellular signal transduction triggered by P-cadherin involves activation of Rac1 and Cdc42 Rho GTPases [33], but it has been found that Rac1 and Cdc42 activation alone does not fully account for the ability of P-cadherin to suppress anoikis [14]. There is evidence that EMT confers anoikis resistance in some cell types [32]. However, we have found that P-cadherin does not alter expression of EMT-promoting transcription factors in EOC cells [14]. We also cannot exclude the possibility that HOXA9 inhibit anoikis by a mechanism that is independent of P-cadherin. Because TGF-β signaling induces EMT [34] and HOXA9 induces TGF-β2 expression in EOC cells [10], HOXA9 might inhibit anoikis by promoting EMT. However, we found that HOXA9 neither represses E-cadherin expression [Figure 3A] nor alters expression of EMT-promoting transcription factors in EOC cells, including those that are induced by TGF-β signaling [10]. Autocrine TGF-β signaling appears to be impaired in EOC cells that express HOXA9 [10] and this impairment could stem from the ability of HOXA9 to block TGF-β-induced Smad-dependent transcription [35]. Collectively, these findings strongly suggest that the ability of HOXA9 to inhibit anoikis in EOC cells is largely attributable to its induction of P-cadherin and the facilitation of EOC cell aggregation by P-cadherin.

The second important rate-limiting step in the progression of EOC is the implantation of floating tumor cells on to peritoneal surfaces. Interactions of EOC cells with peritoneal mesothelial cells and/or the submesothelial extracellular matrix are mediated by various cell surface molecules including CD44 and several integrins [36,37]. We previously identified that HOXA10, a homeoprotein that is expressed in a subset of EOCs, can promote interactions between EOC cells and peritoneal mesothelial cells by inducing expression of αvβ3 integrin [38]. The notion that P-cadherin facilitates tumor-peritoneum interactions is supported by findings that P-cadherin is frequently expressed in malignant peritoneal effusions of EOC patients and is the most predominant type of cadherin in normal peritoneal tissues [11,28,39]. The findings of the present study indicate that the ability of HOXA9 to promote interactions between EOC cells and peritoneal mesothelial cells is primarily mediated by its induction of P-cadherin. Inhibiting P-cadherin in HOXA9+ EOC cells abrogated the stimulatory effect of HOXA9 on mesothelial cell attachment, and reconstitution of P-cadherin in HOXA9-knockdown cells restored attachment capability. Our work also demonstrates that HOXA9 promotes migratory potential of EOC cells via its induction of P-cadherin. The function of P-cadherin in cell migration varies depending on the type of cell. It has been reported that P-cadherin inhibits migration of melanoma and oral squamous cell carcinoma cells [40,41], but promotes migration of pancreatic and bladder cancer cells [33,42]. Our findings support prior reports that P-cadherin stimulates migratory potential of EOC cells [13,14].

Because the precise mechanism of HOXA9 in controlling female reproductive development is not known, it is unclear how its induction of P-cadherin in EOC might be related to its normal developmental function. Cadherin-mediated cell adhesion is essential for maintaining the structure and function of reproductive tissues [43], but mice with targeted disruption of the *Cdh3* or *Hoxa9* genes are fertile [44,45]. The mechanisms that induce HOXA9 expression in EOC are also poorly understood. One possible mechanism is deregulated promoter methylation. *HOXA9* promoter methylation has been detected at significantly lower frequency in advanced-stage EOC than in early-stage tumors [46]. Another possible mechanism is histone modification. Polycomb group (PcG) proteins form complexes that dynamically alter chromatin structure by modifying specific residues in histone tails and PcG-mediated repression is a principal mechanism by which *HOX* gene expression is tightly regulated during development [47]. AKT signaling has been reported to suppress the histone methyltransferase activity of the PcG protein EZH2 [48], and is frequently activated in high-grade, advanced-stage EOCs [49]. It is therefore possible that AKT activation in EOC derepresses *HOXA9* expression by inhibiting EZH2 activity. Interestingly, HOXA9 is not endogenously expressed in the parental SKOV3 cell line but in the aggressive subclone SKOV3ip [Figure 3A]. The expression of HOXA9 in SKOV3ip cells might be attributable to SKOV3ip cells having higher ERBB2 levels than the parental SKOV3 cells [15].

## Conclusion

The present study supports increasing evidence of the functional significance of homeoproteins in dictating the clinical behavior of tumors through controlling transcription of distinct sets of target genes. Because homeoproteins are transcription factors and share tracts of homology, it is challenging to therapeutically target these proteins. However, elucidating the mechanisms of homeoproteins and their downstream effectors in tumors can yield important insights into more effective therapeutic strategies. Inhibiting P-cadherin is particularly promising as a human monoclonal Ab to P-cadherin has been developed and is undergoing clinical trials [50].

## Methods

### Abs and plasmids

Abs to HOXA9 were purchased from Millipore (for Western blot) and Santa Cruz Biotechnology (for chromatin IP). Abs to P-cadherin were purchased from BD Biosciences (for Western blot) and Abcam (for neutralization). Other Abs were as follows: E-cadherin (Invitrogen), N-cadherin, active caspase-3 (BD Biosciences), actin, secondary Abs (Sigma-Aldrich). *HOXA9* cDNA was provided by Corey Largman (Veterans Affairs Medical Center, San Francisco). *CDH3* cDNA and pGFP-V-RS plasmids containing *HOXA9*, *CDH3* and non-targeting shRNAs were purchased from OriGene Technologies.

### Cell culture and transfection

SKOV3ip cell lines that stably express non-targeting and *HOXA9* shRNAs have been previously described [10]. The parental SKOV3 cell line was purchased from American Type Culture Collection. Cell lines were authenticated by short tandem repeat analysis performed by the MD Anderson Cancer Center Characterized Cell Line Core Facility. SKOV3ip and SKOV3 cells were cultured in McCoys 5A medium (Invitrogen) supplemented with 10% FBS and penicillin-streptomycin. Cells were transfected by using Lipofectamine 2000 reagent (Invitrogen) and selected by the addition of puromycin (0.5 μg/ml). Primary cultures of normal human omental mesothelial cells have been previously described [51] and were provided by Ernst Lengyel (University of Chicago, Chicago, IL).

### Western blot and qRT-PCR analyses

Cell lysates were prepared by using M-PER buffer (Pierce Biotechnology), separated by SDS-PAGE and transferred to polyvinylidene difluoride membranes (GE Healthcare). *CDH3* transcripts were analyzed by using SYBR®Green qPCR Master Mix (SABiosciences) and the following primers: forward: 5′-CAGGTGCTGAACATCACGGACA-3′, reverse: 5′-CTTCAGGGACAAGACCACTGTG-3′. *RPL32* transcript levels were used as controls for normalization and were detected by using the following primers: forward: 5′-ACAAAGCACATGCTGCCCAGTG-3′ reverse: 5′- TTCCACGATGGCTTTGCGGTTC-3′.

### Chromatin IP

Chromatin IP assays were performed by using the EZ-ChIP Assay kit (Millipore). Sheared chromatin was incubated overnight with 1 μg of HOXA9 Ab. DNA was purified from precipitated complexes. Fragments of the human *CDH3* promoter were amplified by using the following primers: for Site A (150 bp), forward: 5′-CCCCCA CCCTCGCAACGCAAGCAA-3′, reverse: 5′-TGGGATTACAGGCGTGAGAAACG-3′; for Site B (104 bp), forward: 5′-GCTGTGTGCCAGATATGATGTTTAG-3′, reverse: 5′-CTGTCAAATGGGGCTGTTATGATC-3′. A 166 bp fragment of the *GAPDH* gene was amplified as an irrelevant control by using the following primers: forward: 5′-TACTAGCGGTTTTACGGGCG-3′, reverse: 5′-TCGAACAGGAGGAGCAGAGAGCGA-3′.

### Cell death assays

EOC cells were seeded in 96-well plates (1 × 10^4^ cells per well) that were coated with polyHEMA (Sigma-Aldrich) to block cell attachment to substratum as previously described [38]. Where indicated, EOC cells were incubated with neutralizing P-cadherin Ab or with control IgG at a final concentration of 10 μg/ml. At 3 days thereafter, cell morphology was viewed by phase-contrast light microscopy. Cells were evaluated for cell death by staining with 7AAD (Sigma-Aldrich) and with Ab to active caspase-3. Staining was detected by flow cytometry (FACS Calibur, BD Biosciences). Cell death was also evaluated by assaying mono- and oligo- nucleosomes in cell lysates by using the Cell Death Detection ELISA kit (Roche). Three independent experiments were performed for each assay.

### Cell attachment assays

Single cell suspensions of GFP-expressing EOC cells were seeded in 96-well plates (1.5 × 10^4^ per well) containing confluent monolayers of normal omental mesothelial cells as previously described [38]. At 1 hr after seeding of EOC cells, wells were washed with culture medium to remove unattached EOC cells. Attached EOC cells were viewed by fluorescence microscopy using a fluorescein filter. Three independent experiments were performed in which attached cells were counted in three random 100× microscopic fields in each experiment.

### Cell migration assays

EOC cells were seeded as single cell suspensions in the upper chamber of 24-well transwell chambers (BD Biosciences) (5 × 10^4^ cells per well). At 6 h thereafter, migrating cells were stained with Giemsa solution. Three independent experiments were performed in which migrating cells were counted in three random 100x microscopic fields in each experiment.

### Mouse i.p. xenograft studies

Four-week-old female nude mice (purchased from the National Cancer Institute, Frederick, MD) were inoculated i.p. with 2 × 10^6^ cells of GFP-expressing EOC lines (n = 5 mice per group). Mice were euthanized by CO_2_ asphyxiation at time points indicated in the figure legends. GFP-expressing tumor implants were visualized under a Leica MZML III stereomicroscope equipped with a mercury lamp power supply and GFP filter set. Formalin-fixed, paraffin-embedded tissue sections were stained with hematoxylin-eosin (HE) and analyzed under a light microscope. Invasive depth of implants was measured using a stage micrometer slide in five random microscopic fields of tissue sections of each mouse at the magnifications indicated in the figure legends. Floating cells were collected from peritoneal fluid, stained with Hoechst dye and viewed by fluorescence microscopy. Floating cells were also stained with 7AAD and cell clusters gently disaggregated by passing through 35 μm nylon mesh. Immediately thereafter, 7AAD staining was analyzed by flow cytometry within the gated population of GFP + tumor cells.

### Bioinformatic analysis

Gene expression data of EOC cases from the TCGA project (n = 567) were downloaded from the TCGA data portal site (http://tcga-data.nci.nih.gov/tcga/). Where there were multiple probe sets for an individual gene, the mean value for the given gene for each case was used. Patients were stratified according to the level of *HOXA9* expression in tumors, where *HOXA9* transcript levels were defined as High (≥ upper quartile) and Low (≤ lower quartile) as previously described [10].

### Statistical analysis

Statistical analysis was performed by using STATISTICA6 software (StatSoft Inc.). Statistical significance of data of *in vitro* and *in vivo* assays was assessed by unpaired two-tailed Student’s *t*-test. Data represent mean ± s.d. Significance of differences in gene expression between groups of patients was assessed by Mann–Whitney *U*-test. *P* values of < 0.05 were considered significant.

## Abbreviations

7AAD: 7-amino actinomycin D; Ab: Antibody; EOC: Epithelial ovarian cancer; ECAD: E-cadherin; HE: Hematoxylin-eosin; NCAD: N-cadherin; PCAD: P-cadherin; PcG: Polycomb group; PolyHEMA: Poly (2-hydroxyethyl methacrylate); shRNAs: Short hairpin RNAs; TGF: Transforming growth factor.

## Competing interest

The authors have declared that no conflict of interest exists.

## Authors’ contribution

SYK and HN designed and performed experiments and wrote the manuscript. Both authors read and approved the final manuscript.

## Acknowledgements

This work was supported by Cancer & Prevention Research Institute of Texas grant RP120390 (to H. Naora) and U.S. National Institutes of Health grant CA141078 (to H. Naora). We thank Nicolas Barengo for technical assistance and members of the Naora laboratory for helpful discussions.

## Supplementary Material

Additional file 1: Figure S1 Invasiveness of EOC cells in i.p. xenograft models. Female nude mice (n=5 per group) were inoculated i.p. with equivalent numbers of cells (2 × 10^6^) of SKOV3ip lines and sacrificed at 3 weeks thereafter. Invasive depth of implants on the bowel, diaphragm and peritoneal cavity wall was measured in five random microscopic fields of HE-stained tissue sections of each of these sites in each mouse. An average invasive depth was calculated for each site of each mouse. **(A)** Depth of superficial bowel serosa invasion (evaluated at 200× magnification) and representative examples of HE-stained sections. Bar, 50 μm. **(B)** Depth of invasion of diaphragmatic implants into adjacent muscle (evaluated at 100x magnification). Representative examples of HE-stained sections of diaphragmatic implants are shown in Figure 1D. **(C)** Invasive depth of implants on the peritoneal cavity wall (evaluated at 100× magnification) and representative examples of HE-stained sections. Bar, 100 μm. Click here for file

Additional file 2: Figure S2 Quantification of protein levels. Protein levels were evaluated by measuring intensity of bands on Western blots shown in Figures 3A, 4A and 5A using the TINA 20 program (Raytest). **(A)** Levels of an individual protein in SKOV3ip lines transfected with non-targeting and *HOXA9* shRNAs are expressed relative to its level in the empty vector control SKOV3ip line. Levels of an individual protein in the HOXA9-transfected SKOV3-Par line are expressed relative to its level in the empty vector control SKOV3-Par line. **(B)** Levels of P-cadherin in HOXA9-transfected SKOV3-Par lines that were co-transfected with no shRNA, non-targeting shRNA or *CDH3* shRNAs are expressed relative to its level in the empty vector control SKOV3-Par line. **(C)** Levels of P-cadherin in HOXA9-knockdown SKOV3ip cells and HOXA9-knockdown SKOV3ip cells that stably expressed P-cadherin are expressed relative to its level in SKOV3ip cells expressing non-targeting shRNA. Click here for file

Additional file 3: Figure S3 Effects of P-cadherin Ab on HOXA9-overexpressing EOC cells. Cells of vector-control and HOXA9-transfected SKOV3-Par lines were incubated as suspension cultures in polyHEMA-coated plates for 3 days with the addition of neutralizing P-cadherin Ab or control IgG. **(A)** Cell morphology viewed by phase-contrast microscopy. Bar 50 μm. **(B)** Cell death was evaluated by assaying mono- and oligo- nucleosomes in cell lysates by ELISA. Shown are mean + sd values of three independent experiments. Click here for file

## References

[B1] Siegel R Naishadham D Jemal A Cancer statistics, 2013 CA Cancer J Clin 2013 63 11 30 23335087 10.3322/caac.21166

[B2] Naora H Montell DJ Ovarian cancer metastasis: integrating insights from disparate model organisms Nat Rev Cancer 2005 5 355 366 15864277 10.1038/nrc1611

[B3] Lengyel E Ovarian cancer development and metastasis Am J Pathol 2010 177 1053 1064 20651229 10.2353/ajpath.2010.100105 PMC2928939

[B4] Sodek KL Murphy KJ Brown TJ Ringuette MJ Cell-cell and cell-matrix dynamics in intraperitoneal cancer metastasis Cancer Metastasis Rev 2012 31 397 414 22527451 10.1007/s10555-012-9351-2 PMC3350631

[B5] Burleson KM Casey RC Skubitz KM Pambuccian SE Oegema TR Jr Skubitz AP Ovarian carcinoma ascites spheroids adhere to extracellular matrix components and mesothelial cell monolayers Gynecol Oncol 2004 93 170 181 15047232 10.1016/j.ygyno.2003.12.034

[B6] Abate-Shen C Deregulated homeobox gene expression in cancer: cause or consequence? Nat Rev Cancer 2002 2 777 785 12360280 10.1038/nrc907

[B7] Samuel S Naora H Homeobox gene expression in cancer: insights from developmental regulation and deregulation Eur J Cancer 2005 41 2428 2437 16199152 10.1016/j.ejca.2005.08.014

[B8] Pearson JC Lemons D McGinnis W Modulating Hox gene functions during animal body patterning Nat Rev Genet 2005 6 893 904 16341070 10.1038/nrg1726

[B9] Taylor HS Vanden Heuvel GB Igarashi P A conserved Hox axis in the mouse and human female reproductive system: late establishment and persistent adult expression of the Hoxa cluster genes Biol Reprod 1997 57 1338 1345 9408238 10.1095/biolreprod57.6.1338

[B10] Ko SY Barengo N Ladanyi A Lee JS Marini F Lengyel E Naora H HOXA9 promotes ovarian cancer growth by stimulating cancer-associated fibroblasts J Clin Invest 2012 122 3603 3617 22945634 10.1172/JCI62229 PMC3461910

[B11] Patel IS Madan P Getsios S Bertrand MA MacCalman CD Cadherin switching in ovarian cancer progression Int J Cancer 2003 106 172 177 12800191 10.1002/ijc.11086

[B12] Quattrocchi L Green AR Martin S Durrant L Deen S The cadherin switch in ovarian high-grade serous carcinoma is associated with disease progression Virchows Arch 2011 459 21 29 21509572 10.1007/s00428-011-1082-1

[B13] Cheung LW Leung PC Wong AS Cadherin switching and activation of p120 catenin signaling are mediators of gonadotropin-releasing hormone to promote tumor cell migration and invasion in ovarian cancer Oncogene 2010 29 2427 2440 20118984 10.1038/onc.2009.523

[B14] Usui A Ko SY Barengo N Naora H P-cadherin promotes ovarian cancer dissemination through tumor cell aggregation and tumor-peritoneum tnteractions Mol Cancer Res 2014 12 504 513 24448686 10.1158/1541-7786.MCR-13-0489 PMC3989397

[B15] Yu D Wolf JK Scanlon M Price JE Hung MC Enhanced c-erbB-2/neu expression in human ovarian cancer cells correlates with more severe malignancy that can be suppressed by E1A Cancer Res 1993 53 891 898 8094034

[B16] Pasca di Magliano M Hebrok M Hedgehog signalling in cancer formation and maintenance Nat Rev Cancer 2003 3 903 911 14737121 10.1038/nrc1229

[B17] Klaus A Birchmeier W Wnt signalling and its impact on development and cancer Nat Rev Cancer 2008 8 387 398 18432252 10.1038/nrc2389

[B18] Ranganathan P Weaver KL Capobianco AJ Notch signalling in solid tumours: a little bit of everything but not all the time Nat Rev Cancer 2011 11 338 351 21508972 10.1038/nrc3035

[B19] Care A Silvani A Meccia E Mattia G Stoppacciaro A Parmiani G Peschle C Colombo MP HOXB7 constitutively activates basic fibroblast growth factor in melanomas Mol Cell Biol 1996 16 4842 4851 8756643 10.1128/mcb.16.9.4842 PMC231486

[B20] Coletta RD Christensen K Reichenberger KJ Lamb J Micomonaco D Huang L Wolf DM Muller-Tidow C Golub TR Kawakami K Ford HL The Six1 homeoprotein stimulates tumorigenesis by reactivation of cyclin A1 Proc Natl Acad Sci U S A 2004 101 6478 6483 15123840 10.1073/pnas.0401139101 PMC404070

[B21] Trinh BQ Barengo N Naora H Homeodomain protein DLX4 counteracts key transcriptional control mechanisms of the TGF-beta cytostatic program and blocks the antiproliferative effect of TGF-beta Oncogene 2011 30 2718 2729 21297662 10.1038/onc.2011.4 PMC3116964

[B22] Micalizzi DS Wang CA Farabaugh SM Schiemann WP Ford HL Homeoprotein Six1 increases TGF-beta type I receptor and converts TGF-beta signaling from suppressive to supportive for tumor growth Cancer Res 2010 70 10371 10380 21056993 10.1158/0008-5472.CAN-10-1354 PMC3072046

[B23] Yu Y Davicioni E Triche TJ Merlino G The homeoprotein six1 transcriptionally activates multiple protumorigenic genes but requires ezrin to promote metastasis Cancer Res 2006 66 1982 1989 16488997 10.1158/0008-5472.CAN-05-2360

[B24] Daftary GS Troy PJ Bagot CN Young SL Taylor HS Direct regulation of beta3-integrin subunit gene expression by HOXA10 in endometrial cells Mol Endocrinol 2002 16 571 579 11875117 10.1210/mend.16.3.0792

[B25] Zhu R Wong KF Lee NP Lee KF Luk JM HNF1alpha and CDX2 transcriptional factors bind to cadherin-17 (CDH17) gene promoter and modulate its expression in hepatocellular carcinoma J Cell Biochem 2010 111 618 626 20568120 10.1002/jcb.22742

[B26] Gumbiner BM Regulation of cadherin-mediated adhesion in morphogenesis Nat Rev Mol Cell Biol 2005 6 622 634 16025097 10.1038/nrm1699

[B27] Halbleib JM Nelson WJ Cadherins in development: cell adhesion, sorting, and tissue morphogenesis Genes Dev 2006 20 3199 3214 17158740 10.1101/gad.1486806

[B28] Sivertsen S Berner A Michael CW Bedrossian C Davidson B Cadherin expression in ovarian carcinoma and malignant mesothelioma cell effusions Acta Cytol 2006 50 603 607 17152269 10.1159/000326027

[B29] Veatch AL Carson LF Ramakrishnan S Differential expression of the cell-cell adhesion molecule E-cadherin in ascites and solid human ovarian tumor cells Int J Cancer 1994 58 393 399 7519585 10.1002/ijc.2910580315

[B30] Davidson B Berner A Nesland JM Risberg B Berner HS Trope CG Kristensen GB Bryne M Florenes AV E-cadherin and alpha-, beta-, and gamma-catenin protein expression is up-regulated in ovarian carcinoma cells in serous effusions J Pathol 2000 192 460 469 11113863 10.1002/1096-9896(2000)9999:9999<::AID-PATH726>3.0.CO;2-M

[B31] Kang HG Jenabi JM Zhang J Keshelava N Shimada H May WA Ng T Reynolds CP Triche TJ Sorensen PH E-cadherin cell-cell adhesion in ewing tumor cells mediates suppression of anoikis through activation of the ErbB4 tyrosine kinase Cancer Res 2007 67 3094 3105 17409416 10.1158/0008-5472.CAN-06-3259 PMC3906735

[B32] Kumar S Park SH Cieply B Schupp J Killiam E Zhang F Rimm DL Frisch SM A pathway for the control of anoikis sensitivity by E-cadherin and epithelial-to-mesenchymal transition Mol Cell Biol 2011 31 4036 4051 21746881 10.1128/MCB.01342-10 PMC3187352

[B33] Taniuchi K Nakagawa H Hosokawa M Nakamura T Eguchi H Ohigashi H Ishikawa O Katagiri T Nakamura Y Overexpressed P-cadherin/CDH3 promotes motility of pancreatic cancer cells by interacting with p120ctn and activating rho-family GTPases Cancer Res 2005 65 3092 3099 15833838 10.1158/0008.5472.CAN-04-3646

[B34] Thiery JP Acloque H Huang RY Nieto MA Epithelial-mesenchymal transitions in development and disease Cell 2009 139 871 890 19945376 10.1016/j.cell.2009.11.007

[B35] Shi X Bai S Li L Cao X Hoxa-9 represses transforming growth factor-beta-induced osteopontin gene transcription J Biol Chem 2001 276 850 855 11042172 10.1074/jbc.M005955200

[B36] Lessan K Aguiar DJ Oegema T Siebenson L Skubitz AP CD44 and beta1 integrin mediate ovarian carcinoma cell adhesion to peritoneal mesothelial cells Am J Pathol 1999 154 1525 1537 10329605 10.1016/s0002-9440(10)65406-5 PMC1866607

[B37] Heyman L Kellouche S Fernandes J Dutoit S Poulain L Carreiras F Vitronectin and its receptors partly mediate adhesion of ovarian cancer cells to peritoneal mesothelium in vitro Tumour Biol 2008 29 231 244 18781095 10.1159/000152941

[B38] Ko SY Lengyel E Naora H The Mullerian HOXA10 gene promotes growth of ovarian surface epithelial cells by stimulating epithelial-stromal interactions Mol Cell Endocrinol 2010 317 112 119 20036708 10.1016/j.mce.2009.12.025 PMC2814902

[B39] Chen GT Tai CT Yeh LS Yang TC Tsai HD Identification of the cadherin subtypes present in the human peritoneum and endometriotic lesions: potential role for P-cadherin in the development of endometriosis Mol Reprod Dev 2002 62 289 294 12112590 10.1002/mrd.10121

[B40] Van Marck V Stove C Van Den Bossche K Stove V Paredes J Vander Haeghen Y Bracke M P-cadherin promotes cell-cell adhesion and counteracts invasion in human melanoma Cancer Res 2005 65 8774 8783 16204047 10.1158/0008-5472.CAN-04-4414

[B41] Bauer K Dowejko A Bosserhoff AK Reichert TE Bauer RJ P-cadherin induces an epithelial-like phenotype in oral squamous cell carcinoma by GSK-3beta-mediated Snail phosphorylation Carcinogenesis 2009 30 1781 1788 19654099 10.1093/carcin/bgp175

[B42] Mandeville JA Silva Neto B Vanni AJ Smith GL Rieger-Christ KM Zeheb R Loda M Libertino JA Summerhayes IC P-cadherin as a prognostic indicator and a modulator of migratory behaviour in bladder carcinoma cells BJU Int 2008 102 1707 1714 18990147 10.1111/j.1464-410X.2008.08115.x

[B43] Rowlands TM Symonds JM Farookhi R Blaschuk OW Cadherins: crucial regulators of structure and function in reproductive tissues Rev Reprod 2000 5 53 61 10711736 10.1530/ror.0.0050053

[B44] Lawrence HJ Helgason CD Sauvageau G Fong S Izon DJ Humphries RK Largman C Mice bearing a targeted interruption of the homeobox gene HOXA9 have defects in myeloid, erythroid, and lymphoid hematopoiesis Blood 1997 89 1922 1930 9058712

[B45] Radice GL Ferreira-Cornwell MC Robinson SD Rayburn H Chodosh LA Takeichi M Hynes RO Precocious mammary gland development in P-cadherin-deficient mice J Cell Biol 1997 139 1025 1032 9362520 10.1083/jcb.139.4.1025 PMC2139972

[B46] Wu Q Lothe RA Ahlquist T Silins I Trope CG Micci F Nesland JM Suo Z Lind GE DNA methylation profiling of ovarian carcinomas and their in vitro models identifies HOXA9, HOXB5, SCGB3A1, and CRABP1 as novel targets Mol Cancer 2007 6 45 17623056 10.1186/1476-4598-6-45 PMC1964763

[B47] Soshnikova N Duboule D Epigenetic regulation of vertebrate Hox genes: a dynamic equilibrium Epigenetics 2009 4 537 540 19923920 10.4161/epi.4.8.10132

[B48] Cha TL Zhou BP Xia W Wu Y Yang CC Chen CT Ping B Otte AP Hung MC Akt-mediated phosphorylation of EZH2 suppresses methylation of lysine 27 in histone H3 Science 2005 310 306 310 16224021 10.1126/science.1118947

[B49] Yuan ZQ Sun M Feldman RI Wang G Ma X Jiang C Coppola D Nicosia SV Cheng JQ Frequent activation of AKT2 and induction of apoptosis by inhibition of phosphoinositide-3-OH kinase/Akt pathway in human ovarian cancer Oncogene 2000 19 2324 2330 10822383 10.1038/sj.onc.1203598

[B50] Zhang CC Yan Z Zhang Q Kuszpit K Zasadny K Qiu M Painter CL Wong A Kraynov E Arango ME Mehta PP Popoff I Casperson GF Los G Bender S Anderes K Christensen JG VanArsdale T PF-03732010: a fully human monoclonal antibody against P-cadherin with antitumor and antimetastatic activity Clin Cancer Res 2010 16 5177 5188 20829331 10.1158/1078-0432.CCR-10-1343

[B51] Kenny HA Krausz T Yamada SD Lengyel E Use of a novel 3D culture model to elucidate the role of mesothelial cells, fibroblasts and extra-cellular matrices on adhesion and invasion of ovarian cancer cells to the omentum Int J Cancer 2007 121 1463 1472 17546601 10.1002/ijc.22874

