# Supplementary figures and images for: HOXA9 promotes homotypic and heterotypic cell interactions that facilitate ovarian cancer dissemination via its induction of P-cadherin
Source: Mol Cancer. 2014 Jul 14;13:170. doi: 10.1186/1476-4598-13-170 (PMC4105245; doi:10.1186/1476-4598-13-170)

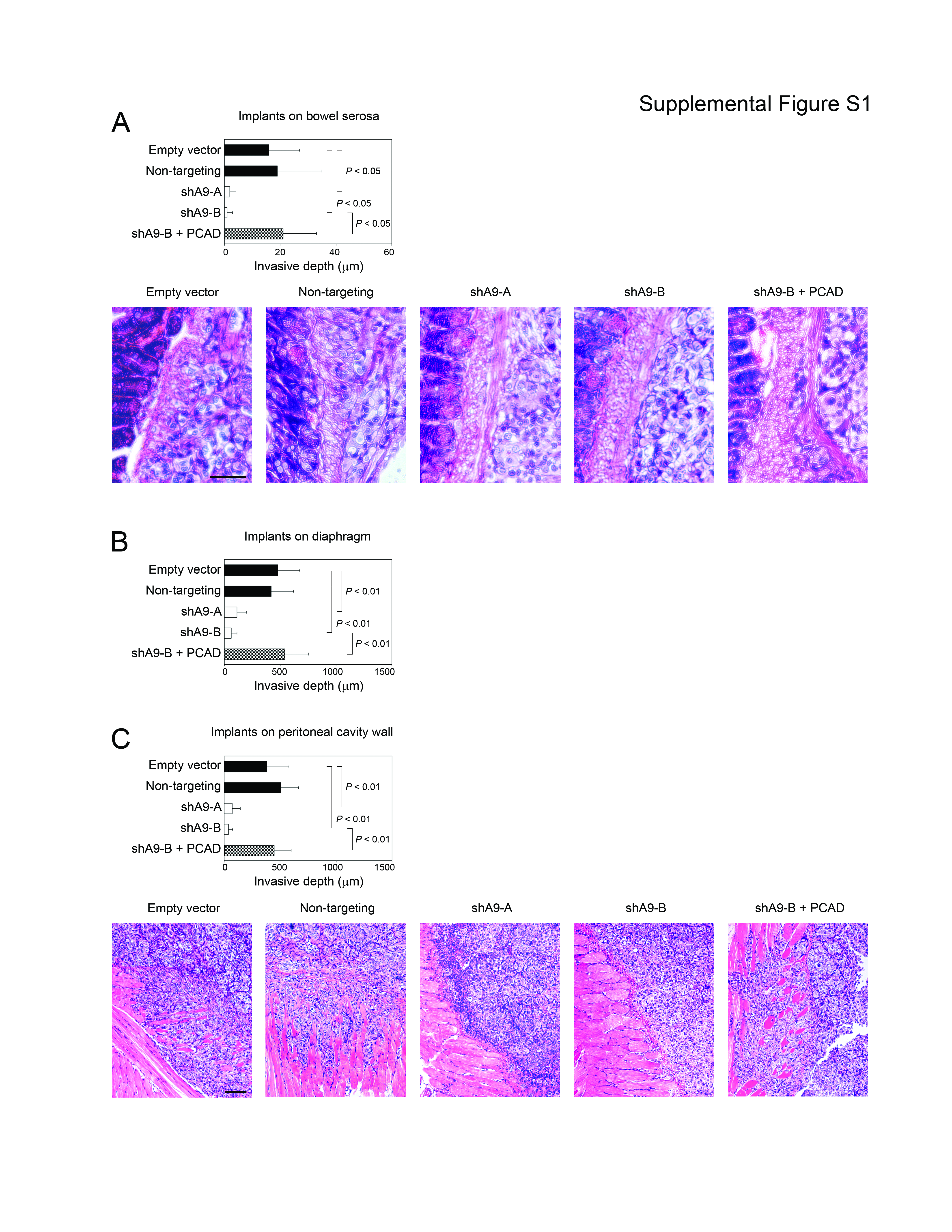

Supplement: Additional file 1: Figure S1 — Invasiveness of EOC cells in i.p. xenograft models. Female nude mice (n=5 per group) were inoculated i.p. with equivalent numbers of cells (2 × 10^6^) of SKOV3ip lines and sacrificed at 3 weeks thereafter. Invasive depth of implants on the bowel, diaphragm and peritoneal cavity wall was measured in five random microscopic fields of HE-stained tissue sections of each of these sites in each mouse. An average invasive depth was calculated for each site of each mouse. (A) Depth of superficial bowel serosa invasion (evaluated at 200× magnification) and representative examples of HE-stained sections. Bar, 50 μm. (B) Depth of invasion of diaphragmatic implants into adjacent muscle (evaluated at 100x magnification). Representative examples of HE-stained sections of diaphragmatic implants are shown in Figure 1D. (C) Invasive depth of implants on the peritoneal cavity wall (evaluated at 100× magnification) and representative examples of HE-stained sections. Bar, 100 μm. [file 1476-4598-13-170-S1.tiff]

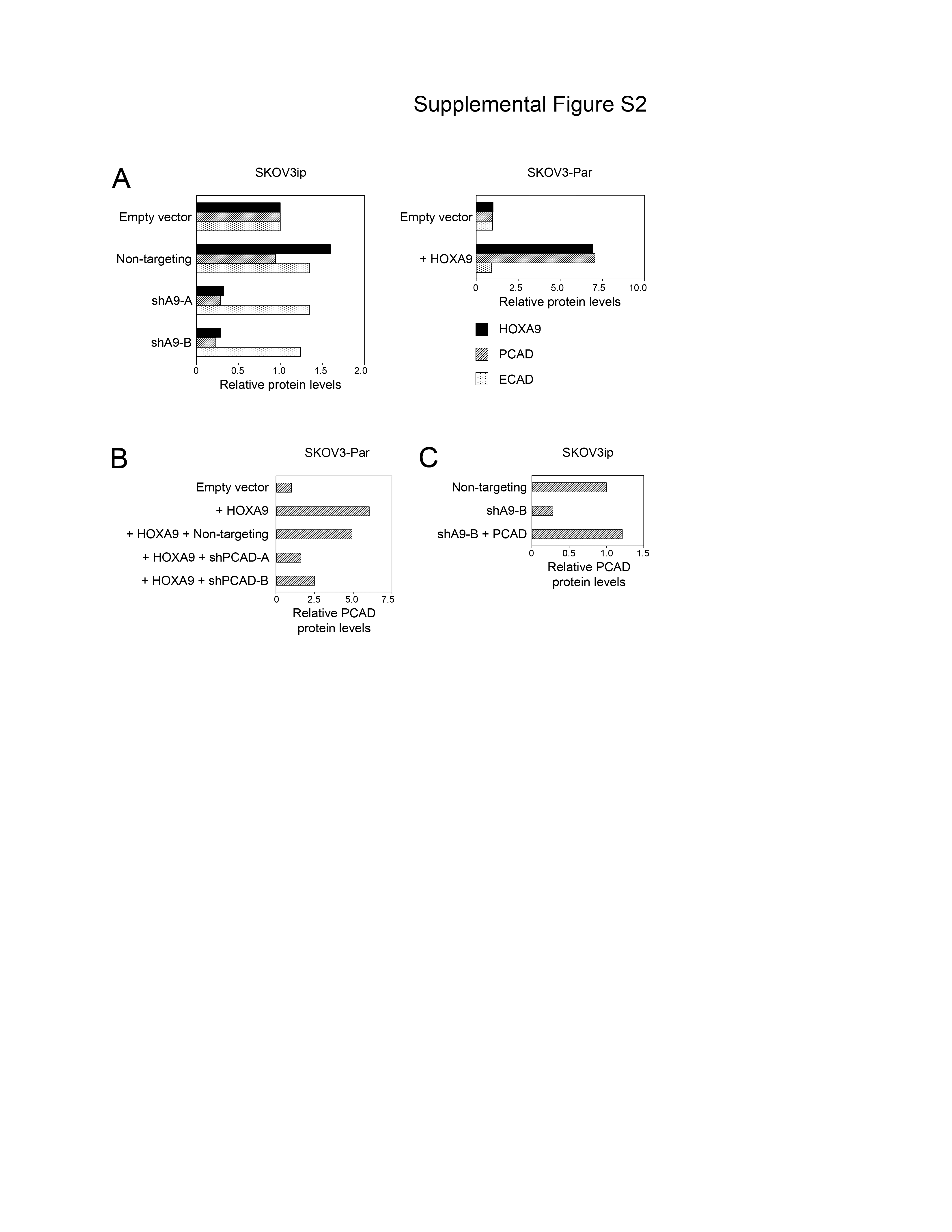

Supplement: Additional file 2: Figure S2 — Quantification of protein levels. Protein levels were evaluated by measuring intensity of bands on Western blots shown in Figures 3A, 4A and 5A using the TINA 20 program (Raytest). (A) Levels of an individual protein in SKOV3ip lines transfected with non-targeting and HOXA9 shRNAs are expressed relative to its level in the empty vector control SKOV3ip line. Levels of an individual protein in the HOXA9-transfected SKOV3-Par line are expressed relative to its level in the empty vector control SKOV3-Par line. (B) Levels of P-cadherin in HOXA9-transfected SKOV3-Par lines that were co-transfected with no shRNA, non-targeting shRNA or CDH3 shRNAs are expressed relative to its level in the empty vector control SKOV3-Par line. (C) Levels of P-cadherin in HOXA9-knockdown SKOV3ip cells and HOXA9-knockdown SKOV3ip cells that stably expressed P-cadherin are expressed relative to its level in SKOV3ip cells expressing non-targeting shRNA. [file 1476-4598-13-170-S2.tiff]

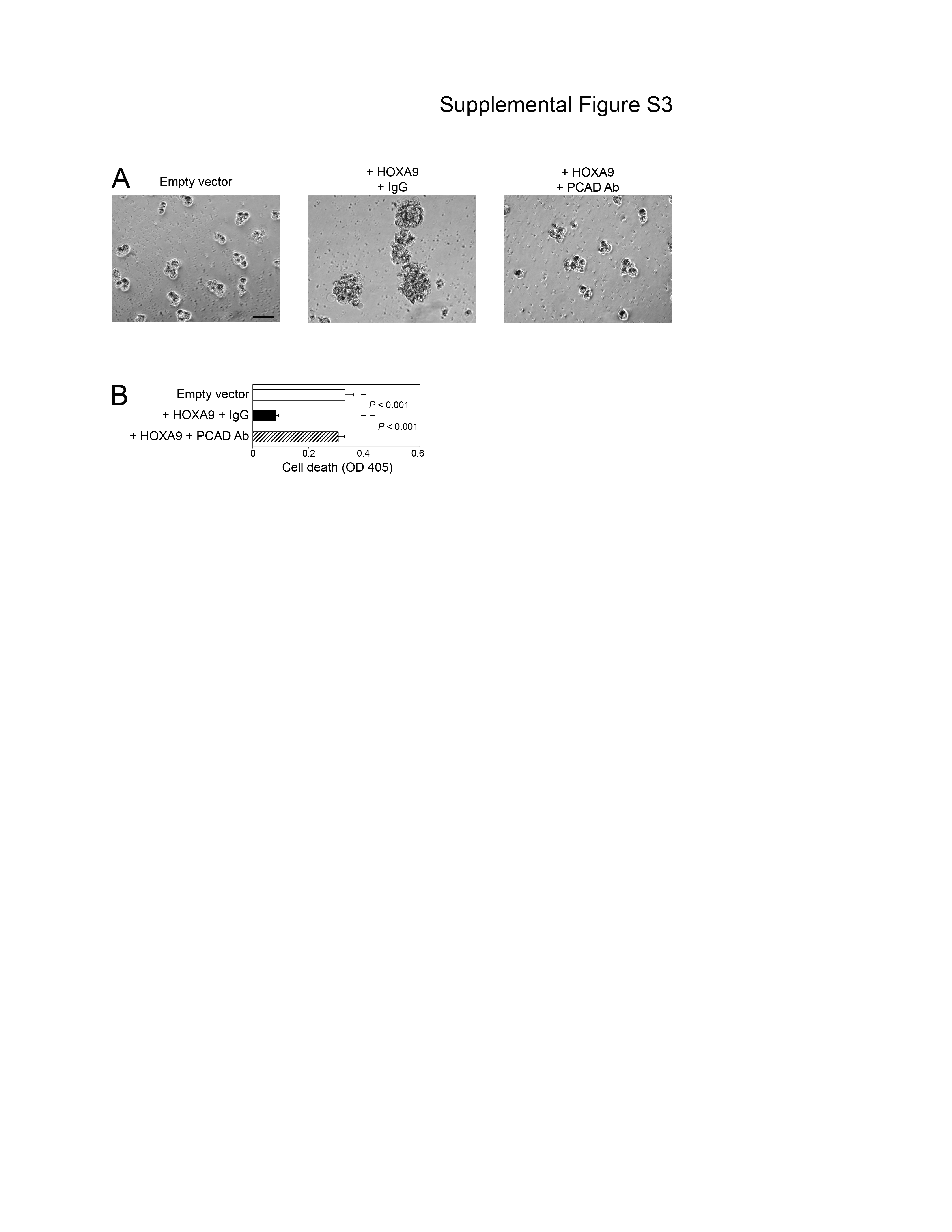

Supplement: Additional file 3: Figure S3 — Effects of P-cadherin Ab on HOXA9-overexpressing EOC cells. Cells of vector-control and HOXA9-transfected SKOV3-Par lines were incubated as suspension cultures in polyHEMA-coated plates for 3 days with the addition of neutralizing P-cadherin Ab or control IgG. (A) Cell morphology viewed by phase-contrast microscopy. Bar 50 μm. (B) Cell death was evaluated by assaying mono- and oligo- nucleosomes in cell lysates by ELISA. Shown are mean + sd values of three independent experiments. [file 1476-4598-13-170-S3.tiff]
